# Automated MRI-based volumetry of basal ganglia and thalamus at the chronic phase of cortical stroke

**DOI:** 10.1007/s00234-020-02477-x

**Published:** 2020-06-17

**Authors:** Cindy Baudat, Bénédicte Maréchal, Ricardo Corredor-Jerez, Tobias Kober, Reto Meuli, Patric Hagmann, Patrik Michel, Philippe Maeder, Vincent Dunet

**Affiliations:** 1 Department of Diagnostic and Interventional Radiology, Lausanne University Hospital and University of Lausanne, Rue du Bugnon 46, CH-1011 Lausanne, Switzerland; 2 Advanced Clinical Imaging Technology, Siemens Healthcare AG, Lausanne, Switzerland; 3 Stroke Center, Neurology Service, Department of Clinical Neurosciences, Lausanne University Hospital and University of Lausanne, Lausanne, Switzerland

**Keywords:** Basal ganglia, Brain morphometry, Magnetic resonance imaging, Stroke, Thalamus

## Abstract

**Purpose:**

We aimed at assessing the potential of automated MR morphometry to assess individual basal ganglia and thalamus volumetric changes at the chronic phase after cortical stroke.

**Methods:**

Ninety-six patients (mean age: 65 ± 18 years, male 55) with cortical stroke at the chronic phase were retrospectively included. Patients were scanned at 1.5 T or 3 T using a T1-MPRAGE sequence. Resulting 3D images were processed with the MorphoBox prototype software to automatically segment basal ganglia and thalamus structures, and to obtain *Z* scores considering the confounding effects of age and sex. Stroke volume was estimated by manual delineation on T2-SE imaging. *Z* scores were compared between ipsi- and contralateral stroke side and according to the vascular territory. Potential relationship between *Z* scores and stroke volume was assessed using the Spearman correlation coefficient.

**Results:**

Basal ganglia and thalamus volume *Z* scores were lower ipsilaterally to MCA territory stroke (*p* values < 0.034) while they were not different between ipsi- and contralateral stroke sides in non-MCA territory stroke (*p* values > 0.37). In MCA territory stroke, ipsilateral caudate nucleus (rho = − 0.34, *p* = 0.007), putamen (rho = − 0.50, *p* < 0.001), pallidum (rho = − 0.44, *p* < 0.001), and thalamus (rho = − 0.48, *p* < 0.001) volume *Z* scores negatively correlated with the cortical stroke volume. This relation was not influenced by cardiovascular risk factors or time since stroke.

**Conclusion:**

Automated MR morphometry demonstrated atrophy of ipsilateral basal ganglia and thalamus at the chronic phase after cortical stroke in the MCA territory. The atrophy was related to stroke volume. These results confirm the potential role for automated MRI morphometry to assess remote changes after stroke.

## Introduction

Stroke is the second largest cause of mortality and the second most common cause of disability-adjusted life-years worldwide, after ischemic heart disease [1, 2]. The World Health Organization estimated that 13.7 million people suffered from a stroke in 2016, of which 5.5 million died. After a stroke, brain plasticity manifests itself as remote changes of the brain, of which some reflect interconnection degeneration and others could counterbalance stroke-induced deficits [3–5]. Identification and quantification of remote changes of the brain are thus of interest in order to find imaging markers for therapy efficacy assessment at the patient level.

In the past decades, automated morphometry of brain magnetic resonance imaging (MRI) has emerged and currently represents a promising tool in clinical practice. Some studies based on morphometric techniques using MRI images led to the better understanding of the mechanisms underlying brain plasticity and functional recovery [6–9]. However, the techniques used for morphometry remain mainly based on group comparisons and are complex to implement in daily practice. These imaging techniques are likely to mask some of the inter-subject variabilities essential to the evaluation of individual brain plasticity. The automated brain MRI segmentation prototype software called MorphoBox has recently been developed to provide volumetric data at the patient level [10]. The measurements obtained with this fully automated algorithm have demonstrated excellent correlation with those obtained with other validated algorithms used for brain morphometry, including FreeSurfer and Statistical Parametric Mapping [10]. It has also shown similar or better performance for the individual classification of patients with cognitive impairment [10] as well as to differentiate patients with Parkinson’s disease from patients with vascular parkinsonism based on their individual brain morphometry [11]. Basal ganglia and thalamus are key anatomical and functional nodes for tracts coming from or going to the cortex, and are thus particularly exposed to remote changes after a cortical injury, such as stroke [4]. Nevertheless, the feasibility and utility of assessing remote changes in basal ganglia and thalamus after cortical stroke with MorphoBox remain to be demonstrated.

The main goal of this work was therefore to evaluate the potential of automated brain MRI morphometry for the detection of volume changes in basal ganglia and thalamus, in patients with chronic stroke. Secondary aims were to evaluate the impact of stroke side, vascular territory, stroke volume, cardiovascular risk factors, and time since stroke on these changes.

## Methods

### Study design

In this single-center retrospective study, patients referred to the Department of Diagnostic and Interventional Radiology of our institution for a brain MRI between January 2010 and August 2018 with a diagnosis of cortical stroke at the chronic phase were searched on our institutional Radiology Information System and consecutively enrolled. Chronic stroke was defined according to current recommendations as stroke more than 3 weeks old [12]. Inclusion criteria were as follows: age > 18 years, single vascular territory stroke involving cortex, stroke size > 2 cm. Exclusion criteria were as follows: associated acute stroke, posterior fossa stroke, lacunar or basal ganglia-thalamus stroke, history of chronic neurodegenerative brain disease (e.g., Alzheimer’s disease, multiple sclerosis), severe traumatic brain injury, or brain tumor.

For each subject included in the study, the side (right or left) and the vascular territory (anterior cerebral artery [ACA], middle cerebral artery [MCA], or posterior cerebral artery [PCA]) affected by the stroke were collected as well as stroke etiology and cardiovascular risk factors (hypertension, diabetes, smoking, dyslipidemia). All collected data were anonymized to comply with national ethical guidelines. Therefore, patients’ consent was waived, and the study was conducted in accordance with the World Medical Association Declaration of Helsinki.

### MR imaging acquisition

All patients selected according to the study criteria underwent brain MRI (MAGNETOM Aera, Symphony, Skyra, Skyra fit, Prisma fit, Vida or Verio, all Siemens Healthcare, Erlangen, Germany) at 1.5-Tesla (14%) or 3-Tesla (86%) in the Department of Diagnostic and Interventional Radiology of our institution. The MRI protocol included a 3D sagittal T1 magnetization prepared rapid gradient echo (T1-MPRAGE) sequence using the 1.5 T and 3 T Alzheimer’s disease neuroimaging initiative (ADNI) protocols [13–15] and a 2D axial T2 spin echo (T2-SE) sequence (TR 5000 ms, TE 77 ms, flip angle 150°, matrix 256 × 240, slice thickness 3 mm, slice number 43, voxel size 0.5 × 0.5 × 3 mm^3^).

### MR imaging analysis

**Fig. 1:**
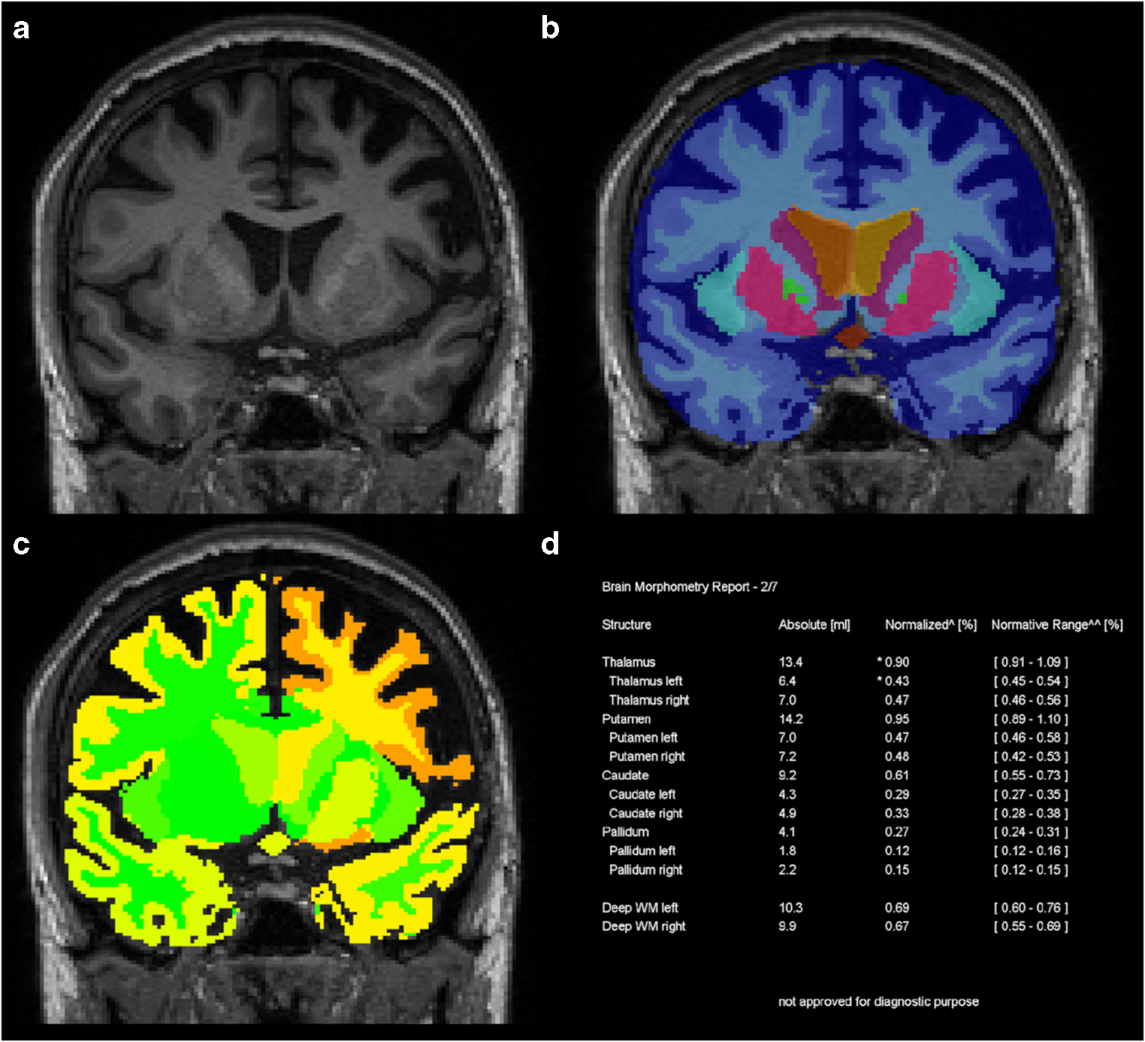
Exemplary segmentation of a MPRAGE sequence with the MorphoBox prototype. From the T1 image (**a**), the algorithm extracts the total brain volume and then segments the different structures (**b**) to obtain a map of the Z-scores (**c**) in comparison with a population of healthy subjects considering age and sex. The final morphometric ratio normalized by the total intracranial volume appears in the form of a Table (**d**) showing atrophy of the left basal ganglia and thalamus in this case of a left MCA stroke (frontal atrophy of the gray and white matter is also seen in orange on image **c**)

MRI analysis was independently performed for T1-MPRAGE and T2-SE sequences. The MPRAGE sequence of each participant was segmented using the automated MorphoBox prototype software [10] to obtain absolute volumes in milliliters (mL) of the following structures for each individual: total intracranial volume (TIV); right and left basal ganglia and thalamus (Fig. 1). These volumes were also expressed as percentage (%) of the TIV and compared to volumes obtained in a population of healthy control subjects included in the MorphoBox tool. This control population corresponds to a subset of healthy subjects included in the ADNI study [15, 16] with additional younger healthy subjects added from in-house acquisitions following ADNI guidelines. In total, it consists of 303 healthy controls (mean age 66 ± 19 years, age range 19–90 years, 51% of healthy male) who underwent 3-Tesla brain MRI using the standardized ADNI protocol. Normative ranges were calibrated on this population using a log-linear regression model, taking into account the confounding effects of age and sex as covariates. Deviations from normative ranges of individual volume estimates were assessed by *Z* scores. For every patients, voxel size in cubic millimeters was recorded, as well as two global contrast-to-noise measurements computed by MorphoBox as image segmentation by-products, respectively between gray matter and white matter (CNRgw) and between gray matter and cerebrospinal fluid (CNRgc).

**Fig. 2:**
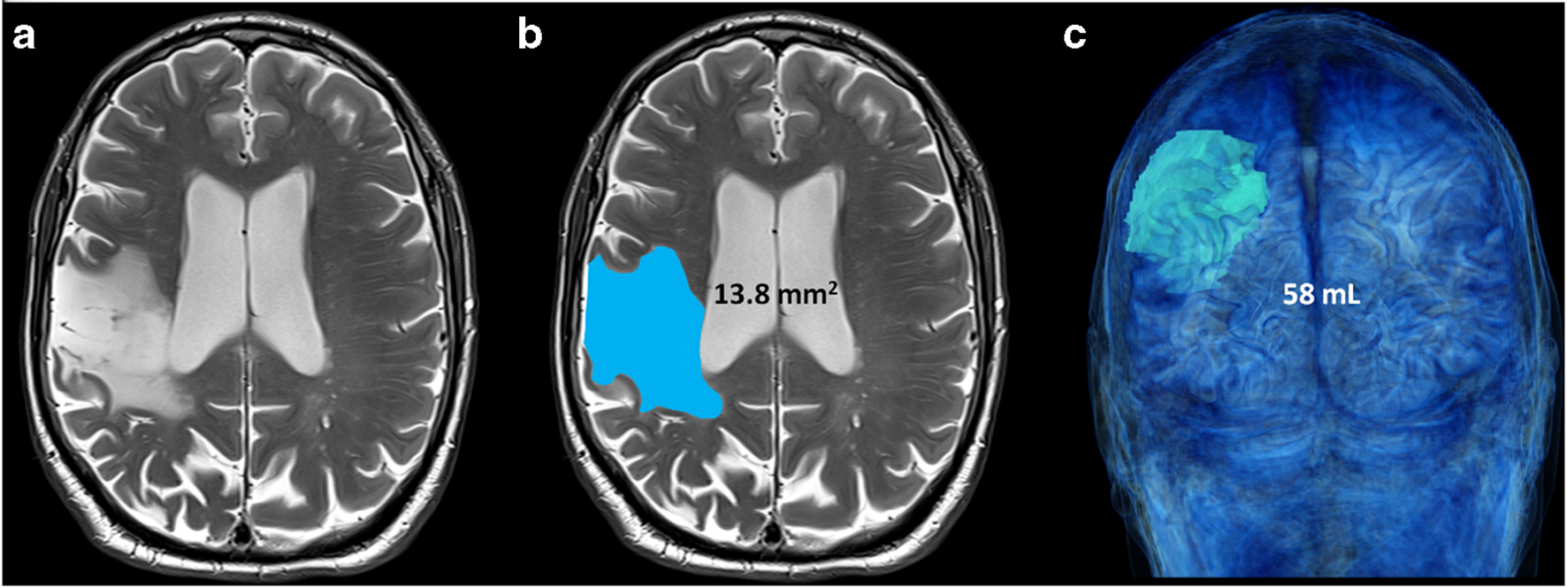
Illustration of the manual segmentation of a right sylvian cortical stroke. On the T2-SE sequence in axial section (**a**), the surface of the cortical stroke was delimited section by section (**b**) and then multiplied by the nominal section thickness to obtain the volume of the stroke, visible on the volume rendering image (**c**)

Axial T2-SE images were analyzed using Carestream software (Version 12.1.6.0117, Carestream Health, Toronto, Canada). The volume of cerebral infarction was manually delineated by a single neuroradiologist with 10 years of experience in brain imaging. A region of interest (ROI) was drawn on each axial section on which the cerebral infarction was visible. The sum of the ROI areas in square millimeters was then multiplied by the nominal slice thickness in millimeters to obtain an estimate of the volume of cerebral infarction in cubic millimeters and in milliliters (Fig. 2). This measure was performed twice for 20 patients randomly chosen to assess estimation reliability. Fazekas score was also recorded to take into account white matter hyperintense lesions, which are a marker of small vessel disease [17].

### Statistics

Statistical analyses were conducted with STATA software (Version 16.0, StataCorp, College Station, Texas, USA). In order to assess the potential correlation between stroke volume and basal ganglia *Z* scores, we performed a sample size calculation. Considering that we aimed to detect a negative correlation of at least − 0.40 with 80% power using a one-sided 5% level test, we estimated a minimum sample size of 47 patients. Continuous variables (age, structure volumes, and *Z* scores, stroke volumes) were expressed as mean ± standard deviations (SD). Categorical variables (sex, side, and vascular territory affected by the stroke, cardiovascular risk factors) were expressed as numbers or percentages. Two groups were defined according to the vascular territory affected by the stroke: strokes affecting the territory of the MCA versus those affecting other vascular territories (i.e., ACA or PCA), referred as “non-MCA” in the rest of the manuscript. This segregation was chosen because the thalamus, putamen, and pallidum are closely connected to the vascularized regions by the MCA. Stroke location distribution between right and left hemispheres and between vascular territories was compared using Fisher’s exact test. Reliability of stroke volume measurement was assessed using Lin’s test. Absolute volumes of strokes, as well as absolute volumes, relative volumes, and *Z* scores of basal ganglia and thalamus were compared between the ipsilateral and contralateral stroke sides and according to the vascular territory affected by the stroke using the Wilcoxon rank-sum test. Potential relation between basal ganglia and thalamus *Z* scores and stroke volumes was assessed using the non-parametric Spearman’s correlation coefficient (rho) and linear regression analysis. Voxel size, CNRgw, and CNRgc were used as covariates to take into account potential bias due to inter-scan variability. Age and delay from stroke onset to MRI were also used as covariates to take into account inter-subject variability. The value of the rho coefficient was interpreted using the Landis and Koch scale, according to the following categorization: very low correlation if rho < |0.20|; low if rho: |0.21–0.40|; moderate if rho: |0.41–0.60|; strong if rho: |0.61–0.80|, and excellent if rho > |0.80|. Finally, multivariate linear regression analysis was performed to assess the impact of cardiovascular risk factors (age, hypertension, smoking, diabetes, dyslipidemia), Fazekas score, carotid artery stenosis ≥ 50%, and delay from stroke onset on this interrelation. For all statistics, the determined *p* value was considered statistically significant for values < 0.05.

## Results

### Study population

Overall, 98 patients (mean age: 65 ± 18 years, female 42, male 56) met the inclusion and exclusion criteria between January 2010 and August 2018. Patients’ characteristics are summarized in Table 1. Two patients were subsequently excluded from volumetric analysis due to poor image quality impeding the manual delineation of the stroke volume on T2-SE images or automated segmentation of MPRAGE. Basal ganglia, thalamus, and stroke volumes were thus obtained for 96 patients (mean age 65 ± 18 years, female 41, male 55). The mean delay between stroke onset and MRI evaluation was 73.1 ± 93.7 months (range 1.3–427 months).

**Table 1:** Patients’ characteristics

Variables	Median [IQR] or number
Sex (male/female)	56/42
Age (years)	69 [57–78]
Weight (kg)	73.2 [64.2–83.0]
BMI (kg/m^2^)	26.0 [22.9–28.4]
Cardiovascular risk factors	
Smoking (past/present/no)	16/26/56
Hypertension (yes/no)	67/31
Dyslipidemia (yes/no)	63/35
Diabetes (yes/no)	21/77
Fibrillation (yes/no)	14/84
Myocardial infarct (yes/no)	15/83
Peripheral artery disease (yes/no)	8/90
Stroke etiologies	
Atherosclerosis	41
bEmbolic	10
Dissection	5
Vasculitis	6
Unkown	36
Stroke location	
Anterior cerebral artery	14
Middle cerebral artery	62
Posterior cerebral artery	22
Vascular burden	
Fazekas score (0/1/2/3)	20/31/30/17
Carotid artery stenosis ≥ 50% (yes/no)	15*/83

*Ten patients with a MCA stroke and 5 patients with a non-MCA stroke had a carotid artery stenosis ≥ 50%. None had an occlusion

Of the 96 patients retained in the volumetric study, 52 had a right-sided stroke and 44 had a left-sided stroke. Regarding the vascular territory, 13 had a stroke in the territory of the ACA, 61 patients had a stroke affecting the territory of the MCA, and 22 had a stroke in the territory of the PCA. There was no significant difference in stroke distribution regarding the side and vascular territory (*p* = 0.69). The mean stroke volume was 29.7 ± 42.3 mL, with no significant difference according to stroke side or vascular territory (*p* > 0.46). The stroke volume in MCA territory was not significantly different from the stroke volume in non-MCA territory (34.5 ± 51.3 mL versus 21.4 ± 15.4 mL, *p* = 0.15). Lin’s test demonstrated excellent reliability of the stroke volume estimation (Pearson’s rho = 0.999, rho_*c* = 0.996, Cb = 0.997, mean difference − 1 ± 3.5 mL, 95% CI − 7.9–5.9 mL). Time since stroke was similar between patients with MCA and non-MCA stroke (*p* = 0.63).

### Volume of basal ganglia and thalamus

**Fig. 3:**
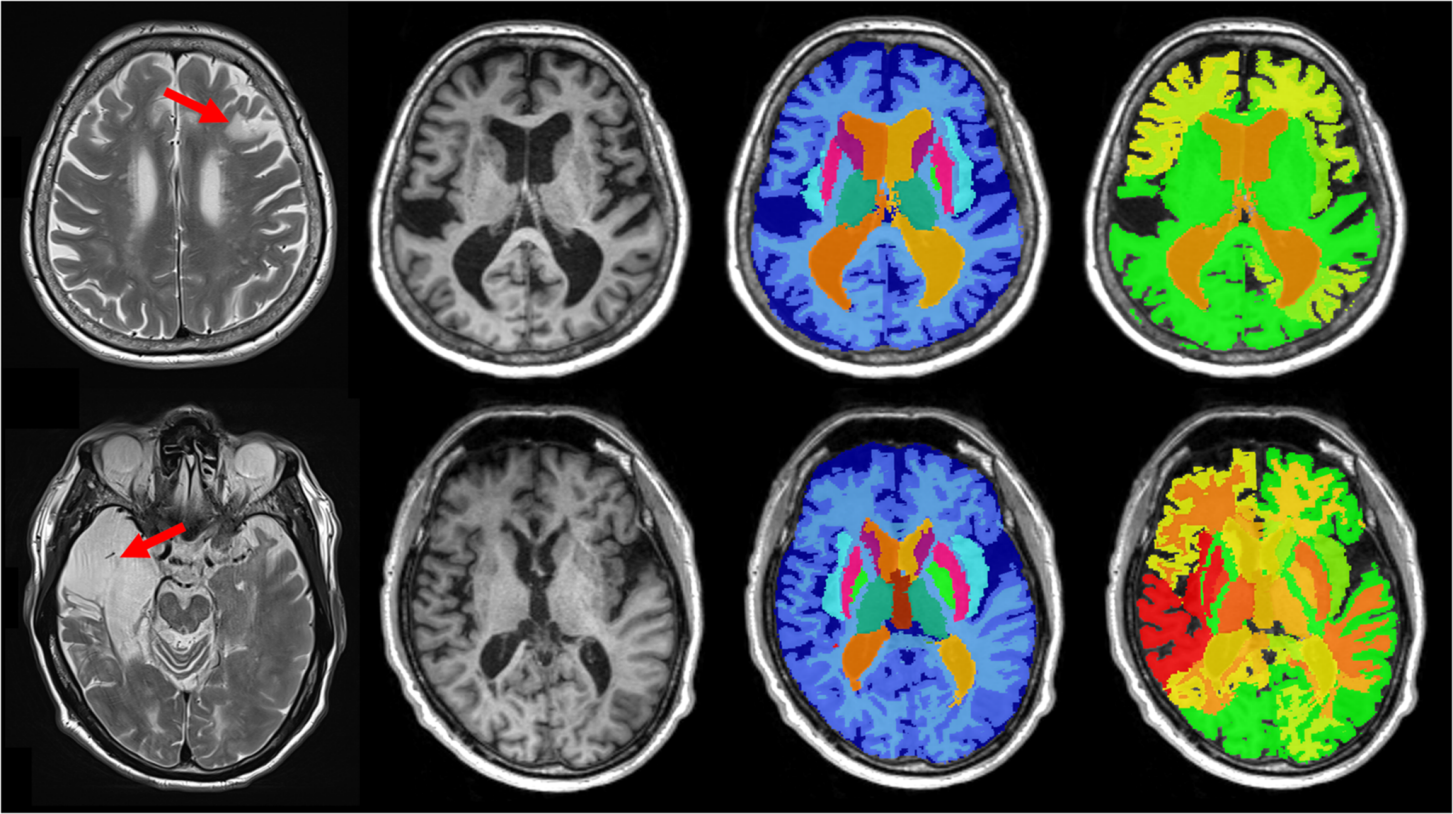
Exemplary segmentation of the MPRAGE sequence in patients with small and large cortical strokes. Small (top row) and large (bottom row) cortical strokes are displayed on axial T2-SE images (left column) along with axial T1 MPRAGE view, segmentation and *Z* score maps. The small cortical stroke measured 8 mL and the large measured 47 mL. Resulting ipsilateral caudate nucleus, putamen, pallidum, and thalamus *Z* scores were 2.10, − 0.07, 1.24, 2.01 and − 1.37, − 5.09, − 3.73, and − 3.34, respectively

MPRAGE scans of all 96 included patients were successfully segmented by the morphometry software. This corresponds to a feasibility of 98% (i.e., 96 of 98 initially enrolled patients). The individual duration of post-processing of the T1-MPRAGE sequences was 2–3 min for each participant (i.e., approximately 5 h in total for all included subjects). Figure 3 displays examples of segmentation in two patients with a small and large cortical stroke, respectively.

**Fig. 4:**
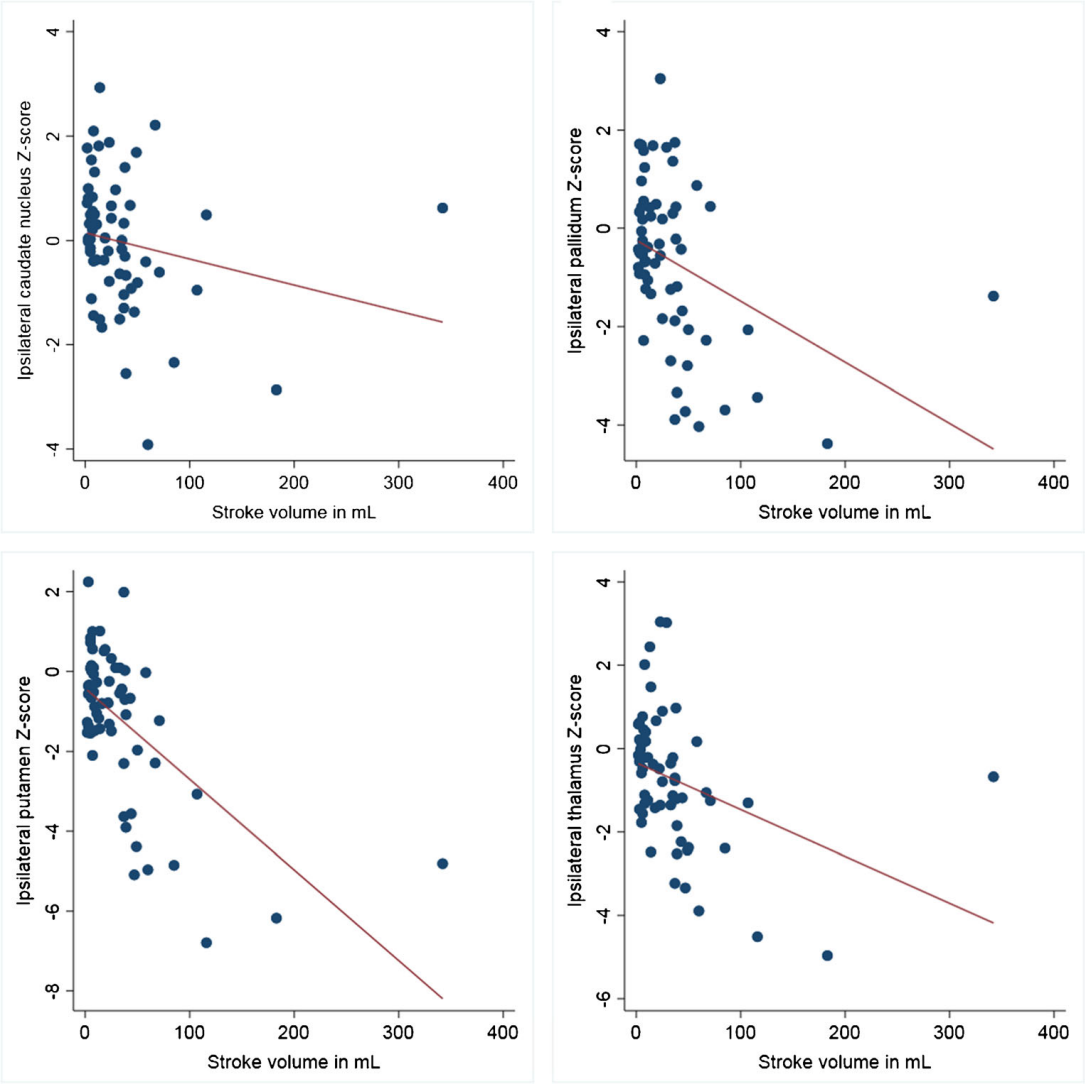
Correlations between stroke volume in the MCA territory and *Z* scores of ipsilateral caudate nucleus, putamen, pallidum, and thalamus volumes

Absolute volumes, relative volumes, and *Z* scores of the basal ganglia and thalamus are summarized in Table 2 according to the ipsilateral or contralateral side of the stroke regardless of the affected vascular territory. Overall, the putamen had a significantly lower absolute volume, relative volume, and *Z* score on the side affected by the stroke. The thalamus had also lower relative volume and *Z* score ipsilaterally to the stroke while the pallidum had a lower *Z* score ipsilaterally to the stroke. The caudate nucleus was not significantly different between the two sides.

**Table 2:** Basal ganglia and thalamus volumes comparison. Absolute volumes, relative volumes, and *Z* scores were compared between ipsi and contralateral sides of the stroke, regardless of the vascular territory, using the Wilcoxon rank sum test. *TIV* total intracranial volume

Volume in mL	Ipsilateral to stroke *n* = 96	Contralateral to stroke *n* = 96	*p* value
Caudate nucleus	4.46 ± 0.87	4.59 ± 0.84	0.14
Putamen	6.08 ± 1.21	6.50 ± 0.90	0.027
Pallidum	1.71 ± 0.34	1.79 ± 0.30	0.15
Thalamus	6.36 ± 1.06	6.67 ± 0.97	0.058
% of the TIV			
Caudate nucleus	0.32 ± 0.05	0.33 ± 0.05	0.13
Putamen	0.44 ± 0.08	0.47 ± 0.06	0.018
Pallidum	0.12 ± 0.02	0.13 ± 0.02	0.077
Thalamus	0.46 ± 0.06	0.48 ± 0.05	0.016
*Z* score			
Caudate nucleus	0.16 ± 1.34	0.48 ± 1.26	0.085
Putamen	− 0.90 ± 1.84	− 0.22 ± 1.29	0.013
Pallidum	− 0.53 ± 1.53	− 0.03 ± 1.17	0.025
Thalamus	− 0.61 ± 1.52	0.09 ± 1.32	0.001

*Z* scores are presented in Table 3 for the MCA and non-MCA territories. For patients with stroke in the MCA territory, the *Z* scores of ipsilateral basal ganglia and thalamus were lower compared to the contralateral side (all *p* < 0.034). In stroke affecting non-MCA territories, the *Z* scores of basal ganglia and thalamus were not significantly different between the ipsilateral and contralateral side (all *p* > 0.37).

**Table 3:** *Z* scores of basal ganglia and thalamus volumes according to vascular territories. *Z* scores were compared between ipsi and contralateral sides of the stroke, according to the vascular territory affected by the stroke, using the Wilcoxon rank sum test. *MCA* middle cerebral artery

Z scores	Ipsilateral to stroke	Contralateral to stroke	*p* value
MCA stroke	*n* = 61	*n* = 61	
Caudate nucleus	− 0.03 ± 1.29	0.50 ± 1.20	0.034
Putamen	− 1.21 ± 1.92	− 0.17 ± 1.26	0.002
Pallidum	− 0.67 ± 1.66	0.10 ± 1.17	0.012
Thalamus	− 0.72 ± 1.59	0.22 ± 1.35	0.001
Non-MCA stroke	*n* = 35	*n* = 35	
Caudate nucleus	0.48 ± 1.40	0.42 ± 1.37	0.90
Putamen	− 0.35 ± 1.57	− 0.31 ± 1.35	0.96
Pallidum	− 0.30 ± 1.27	− 0.25 ± 1.18	0.76
Thalamus	− 0.40 ± 1.37	− 0.13 ± 1.28	0.37

### Correlation between stroke volume and *Z* scores of the basal ganglia and thalamus

Overall, there was a negative correlation between stroke volume and ipsilateral putamen (rho = − 0.39, *p* < 0.001), pallidum (rho = − 0.27, *p* = 0.007), and thalamus (rho = − 0.29, *p* = 0.003) volume *Z* scores but not with contralateral basal ganglia and thalamus volume *Z* scores (all *p* > 0.098). Considering the vascular territory affected by the stroke, there was no significant relation between the stroke volume and *Z* scores of ipsilateral or contralateral basal ganglia and thalamus for non-MCA strokes (all *p* > 0.09). In patients with MCA stroke, the stroke volume was significantly correlated with the *Z* scores of ipsilateral caudate nucleus (rho = − 0.34, *p* = 0.007), putamen (rho = − 0.50, *p* < 0.001), pallidum (rho = − 0.44, *p* < 0.001), and thalamus (rho = − 0.48, *p* < 0.001) (Fig. 4), but not with contralateral *Z* scores (all *p* > 0.28). On multivariate analysis, the putamen (beta = − 0.61, *p* < 0.001), pallidum (beta = − 0.38, *p* = 0.004), and thalamus (beta = − 0.38, *p* = 0.003) *Z* scores were independently correlated with the stroke volume but not with cardiovascular risk factors, Fazekas score, presence of carotid artery stenosis ≥ 50%, or delay from stroke onset (all *p* > 0.24).

## Discussion

The main results of the study can be summarized as follows: (1) Individual automated brain morphometry of patients with cortical stroke was feasible in 98% of our cases; (2) Ipsilateral caudate nucleus, putamen, pallidum, and thalamus were atrophic in patients affected by MCA stroke only; (3) Atrophy of ipsilateral caudate nucleus, putamen, pallidum, and thalamus were negatively correlated with stroke volume. These results indicate that automated brain morphometry in MRI is feasible and can detect remote changes induced by cortical stroke at the chronic phase at the patient level. These changes are influenced by cortical stroke location and extent, but not by cardiovascular risk factors and time since stroke.

### Feasibility of individual morphometry in patients with cortical stroke

Cerebral MR morphometric assessment has undergone significant development over the last 20 years [18]. It allows detecting morphological changes related to congenital or acquired diseases, psychiatric, neurodegenerative, or related to other etiologies. Several morphometric methods are available such as voxel-based analyses, which allow the detection of subtle changes in trophicity, cortical thickness, or the gyri. Nevertheless, these methods are based on a long post-processing of the acquired images and mostly on the comparison between a group of interest and a control group, not giving access in real time to the quantitative analysis in single subjects. The study proceeded to a volume-based morphometry analysis at the patient level rapidly generating an automated volumetric assessment of 45 cerebral structures while focusing on basal ganglia and thalamus. Successful automated segmentation was obtained in 98% of the participants, thus confirming the feasibility as well as the potential of fully automated morphometric approach with tools like MorphoBox in daily clinical practice. In addition, several studies have evaluated the morphological changes of basal ganglia after stroke [3, 8, 9, 19–25]. These studies alternatively used qualitative analyses [21, 22], morphometric methods with manual [19, 23] or semi-automatic delineation [8, 9, 20, 25], overall with a significant risk of segmentation error. None of these studies reported morphometric deviations (i.e., *Z* scores) at the individual level, but only at the group level [8]. We thus report here, to our best knowledge, the first study evaluating morphometric changes at the patient level. This suggests good potential for automated brain morphometry to evaluate individual brain changes in patients with cerebrovascular diseases. While we demonstrated that individual remote morphometric changes in chronic stroke can be assessed by automated segmentation, it remains unknown whether it could be done at the acute or subacute phase after stroke. Its potential to assess the time course of brain changes should hence be evaluated in further longitudinal studies.

### Morphometric changes in basal ganglia and thalamus after cortical stroke

A significant change in basal ganglia volumes from brain reorganization following cortical stroke was expected [26, 27] considering the connection between the cortex and basal ganglia, and confirmed in the present study. Localization of initial cortical stroke significantly influences remote changes of basal ganglia and thalamus. We thus observed atrophy of the caudate nucleus, putamen, pallidum, and thalamus in patients affected by MCA stroke only. This is consistent with several studies that reported morphological changes [21, 22], putamen atrophy [20], or ipsilateral thalamus atrophy [9, 20, 23, 25], indicating thalamic degeneration following proximal occlusion of the MCA. According to Tamura et al. [23], thalamic atrophy at the chronic phase was most likely due to a phenomenon of long-lasting retrograde neuronal degeneration rather than concomitant ischemia during stroke. Indeed, the thalamus and the MCA do not share the same vascular network, as the thalamus is mostly vascularized by the PCA. Regarding the caudate nucleus, results are more controversial, authors alternatively reporting a volume increase of ipsilateral caudate head [8], an absence of significant change in caudate nucleus volume [20], or an atrophy of the ipsilateral caudate nucleus [25]. Nevertheless, comparison with these studies is difficult. In order to specifically evaluate the morphometry of the striatum and thalamus, only patients with purely cortical stroke were included. On the contrary, in most studies published, patients with proximal MCA occlusion or combining all vascular territories were also included; therefore, some of these patients had ischemic lesions of the basal ganglia themselves, which precluded reliable analysis of the volumes of pallidum and putamen, and biased the thalamus and caudate nucleus analyses [19, 21–23, 25]. The first individual automated volumetric analysis of basal ganglia and thalamus at the chronic phase of a purely cortical stroke is reported by our study. While those changes depend on the vascular territory affected by the stroke, it remains unknown whether changes are influenced by some individual factors.

### Impact of stroke volume and cardiovascular risk factors

Finally, beyond stroke location, it was possible to demonstrate that stroke extent had a significant linear impact on the trophicity of the central gray matter ipsilaterally to the affected hemisphere. The volume of cortical stroke negatively correlated with the extent of the morphometric changes of the caudate nucleus, putamen, pallidum, and thalamus in case of an MCA stroke. Tamura et al. [23] have also described that thalamic atrophy appears to be related to the size and location of the infarct, as patients with thalamic atrophy had a large fronto-parietal infarct in their study. In addition, although they did not evaluate the correlation between stroke volume and basal ganglia volume, Yang et al. [25] demonstrated a positive correlation between stroke volume and impaired motor function as well as a negative correlation between ipsilateral thalamus volume and impaired motor function, which suggested a negative relation between infarct and ipsilateral thalamus volumes. While we observed these changes at the chronic phase of a cortical stroke, there is little data on the exact kinetics of occurrence of these changes. Brodtmann et al. [9] suggested that thalamic atrophy could be observed as early as 3 h after the onset of symptoms. In a population of 125 patients who had surgery for resection of a cortical lesion, Kamiya et al. [28] observed transient diffusion restriction of the ipsilateral striatum and thalamus as early as 7 days after the intervention followed by atrophy of these structures; the extension of the anomalies being correlated with the extension of the resection. This indicates the presence of early morphological alterations of the interconnected structures within the striato-thalamo-cortical network ipsilateral to MCA stroke. Finally, we did not find any significant relation between cardiovascular risk factors, Fazekas score, presence of carotid artery stenosis ≥ 50%, or delay from stroke onset, and striato-thalamic *Z* scores after an MCA cortical stroke. Although this suggests that remote changes are independent of cardiovascular risk factors, some other factors such as endothelial function, genetic susceptibility to neuronal degeneration and neovascularization, or other metabolic function such as lactate production [29, 30] may have influenced the individual interrelation between stroke volume and striato-thalamic trophicity. In a transient 1-h murine occlusion model of MCA, Hara et al. [31] demonstrated that atrophy of the thalamus was observed 1 month after the event and could be significantly reduced after administration of a calcium channel blocker, and a glutamate receptor antagonist, which indicated the presence of a trans-synaptic mechanism in the neuronal degeneration of distant structures connected to the ischemic cortical zone (i.e., as the basal ganglia). In humans, the dynamics of basal ganglia alterations after cortical stroke should therefore be the subject of prospective longitudinal studies to determine the optimal time to initiate treatment with a view to stopping, or limiting the mechanisms inducing a cortical stroke neuronal degeneration, such as excitotoxicity, oxidative damage, apoptosis, inflammatory gliosis [27], or to promote mechanisms counteracting this degeneration such as neoangiogenesis [30]. The role of automated brain morphometry to help monitoring potential therapeutic effect could also be assessed.

### Study limitations

Several limitations must be conceded in this study, the first being the small number of patients included with a stroke of the ACA or PCA territories. We did not find significant volume modifications or correlation between stroke volume and basal ganglia *Z* scores, which could be due to lack of statistical power. Results for each of these vascular territories should thus be confirmed in a larger study population. Secondly, our retrospective study lacks longitudinal follow-up data from the acute to the chronic phase. This would be needed to access the exact kinetics of atrophy phenomena as well as to evaluate inter-individual atrophy rate variations. Both might be crucial to determine the optimal time point to treat remote neurodegeneration. Also, prospective longitudinal analysis including structural and functional connectivity mapping could provide a better understanding of potential compensatory networks after initial stroke. Finally, if morphometric changes have been observed, the relation between volumetric changes and patients’ functional recovery should be evaluated; this was out of the scope of the current work.

## Conclusion

Automated MR morphometry demonstrated atrophy of basal ganglia and thalamus at the chronic phase after cortical stroke in the MCA territory, this atrophy being related to stroke volume. These results confirm remote changes of basal ganglia and thalamus after cortical stroke and the potential role for automated MRI volumetry to assess brain plasticity after stroke. Whether automated MRI volumetry could be used to assess acute-subacute remote changes or brain salvage therapies efficacy at the patient level remains unknown and should be evaluated by further longitudinal studies.

## Data Availability

The datasets used during the current study are available from the corresponding author on reasonable request.

## Abbreviations

ACA: Anterior cerebral artery
ADNI: Alzheimer’s disease neuroimaging initiative
CNR: Contrast-to-noise ratio
MCA: Middle cerebral artery
PCA: Posterior cerebral artery
